# Roles of MicroRNAs in Establishing and Modulating Stem Cell Potential

**DOI:** 10.3390/ijms20153643

**Published:** 2019-07-25

**Authors:** Zhenwu Zhang, Lili Zhuang, Chao-Po Lin

**Affiliations:** School of Life Science and Technology, ShanghaiTech University, 230 Haike Rd, Shanghai 201210, China

**Keywords:** microRNA, pluripotency, naïve, primed, embryonic stem cell, trophoblast stem cell, extraembryonic endoderm (XEN) cell

## Abstract

Early embryonic development in mammals, from fertilization to implantation, can be viewed as a process in which stem cells alternate between self-renewal and differentiation. During this process, the fates of stem cells in embryos are gradually specified, from the totipotent state, through the segregation of embryonic and extraembryonic lineages, to the molecular and cellular defined progenitors. Most of those stem cells with different potencies in vivo can be propagated in vitro and recapitulate their differentiation abilities. Complex and coordinated regulations, such as epigenetic reprogramming, maternal RNA clearance, transcriptional and translational landscape changes, as well as the signal transduction, are required for the proper development of early embryos. Accumulated studies suggest that Dicer-dependent noncoding RNAs, including microRNAs (miRNAs) and endogenous small-interfering RNAs (endo-siRNAs), are involved in those regulations and therefore modulate biological properties of stem cells in vitro and in vivo. Elucidating roles of these noncoding RNAs will give us a more comprehensive picture of mammalian embryonic development and enable us to modulate stem cell potencies. In this review, we will discuss roles of miRNAs in regulating the maintenance and cell fate potential of stem cells in/from mouse and human early embryos.

## 1. Introduction

In mammals, early embryonic development can be divided into two stages, the pre-implantation stage and the post-implantation stage. During the preimplantation stage, zygotes go through multiple rounds of cell divisions, forming mature blastocysts which contain three compartments: the epiblast (EPI), the primitive endoderm (PrE), and the trophectoderm (TE), with distinctive developmental potential to give rise to the embryo, the yolk sac, and the placenta, respectively [1,2]. After implantation, the EPI, PrE, and TE are further specified to form embryonic and extraembryonic tissues. Also, shortly after implantation, a crucial type of stem cells, primordial germ cells (PGCs), is specified for the fate of gametes, initiating the next round of the life cycle. The whole process, albeit complex, can be viewed as a cascade of specification, with continuous self-renewal and differentiation of stem cells with various cell fate potential. After decades of efforts, different stem cells from both pre-implantation and post-implantation embryos can be cultured in vitro and still maintain their differentiation potential in vivo. Both animal and cell culture models greatly facilitate our understanding of key events during this earliest stage of life.

Noncoding RNAs can modulate gene expression through different mechanisms [3,4,5]. They are grossly classified by their sizes as short (19–31 nucleotides [nt]), midsize (~20–200 nt), and long (>200 nt) noncoding RNAs. Among short noncoding RNAs, microRNAs (miRNAs), endogenous small-interfering RNAs (endo-siRNAs), and PIWI-interacting RNAs (piRNAs) are the three classes most extensively studied [6]. miRNAs and endo-siRNAs share some common cellular machineries for biogenesis and use similar effector protein complexes for post-transcriptional silencing of specific genes [3]. In mammals, miRNAs are able to regulate almost all biological processes, whereas functions of endo-siRNAs are relatively unknown [4,7]. Substantial evidence demonstrates that miRNAs and endo-siRNAs participate in early embryogenesis in mice. Both in vitro and in vivo models suggest that miRNAs exert biological effects through regulating the self-renewal and differentiation of stem cells in/from early embryos. Since functions of miRNAs in pluripotent stem cells are well-covered elsewhere [8], in this review, we will focus on comparing in vivo and in vitro roles of miRNAs in regulating the potential of stem cells during early embryogenesis in mice and humans.

## 2. Biogenesis of miRNAs and siRNAs

**Figure 1:**
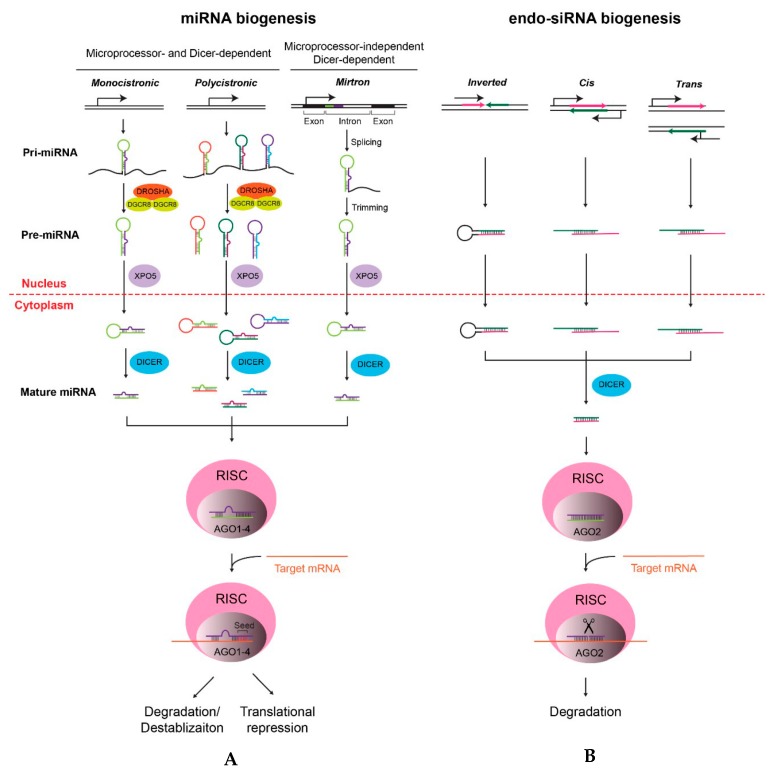
Dicer-dependent biogenesis of miRNAs (**A**) and endo-siRNAs (**B**). (**A**) Biogenesis of miRNAs can be microprocessor- and DICER-dependent (monocistronic and polycistronic miRNAs), or microprocessor-independent and DICER-dependent (mirtrons), with very few exceptions. For the former, monocistronic and polycistronic miRNAs are transcribed by RNA polymerase II as primary miRNAs (pri-miRNAs), which are processed by the microprocessor complex (DROSHA and DGCR8) and exported to the cytoplasm as precursor miRNAs (pre-miRNAs) for the further processing by DICER. Mirtrons, in contrast, are located in the intron and are generated by splicing and trimming that do not need the microprocessor complex to form pre-miRNAs. In both cases, pre-miRNAs need DICER for forming short mature miRNA duplexes. One strand of the duplex is then loaded onto the RNA-interference complex (RISC), where the miRNA recognizes its target mRNA through imperfect base pairing, especially the complementation between the short “seed” sequence of the miRNA and its mRNA targets, performing post-transcriptional silencing on target mRNAs through degradation or translational repression. (**B**) The biogenesis of endo-siRNAs starts with the formation of duplexes from one or two transcripts with complementary sequences. Duplexes are exported to the cytoplasm and processed by DICER as well. Different from miRNAs, endo-siRNAs duplex with their target mRNAs with a higher degree of complementation, inducing the splicer activity of Ago2 for the cleavage of mRNAs, leading to its degradation. Please note that miRNA RISC can be formed with Ago1–4, while the RISC for endo-siRNAs contains Ago2.

MicroRNAs (miRNAs) are short non-coding RNAs, ranging from 22 to 24 nt in length, that repress gene expression at the post-transcriptional level in plants and animals [4]. In animals, miRNAs participate in a variety of biological processes, including the development and diseases [9]. miRNAs are typically transcribed by RNA polymerase II as primary miRNAs (pri-miRNAs), ranging from hundreds to thousands of nucleotides [10] (Figure 1A). Pri-miRNAs can either be monocistronic or polycistronic, encoding multiple miRNAs in the same transcript (Figure 1A). Mammalian pri-miRNAs are methylated by methyltransferase-like 3 (Mettl3) [11] and processed into 60–70 nt precursor miRNAs (pre-miRNAs) by microprocessor complexes, which consist of an RNase III enzyme, Drosha, and an RNA-binding protein, DiGeorge syndrome critical region gene 8 (Dgcr8) [12]. Dgcr8 binds to the pri-miRNA by recognizing an N^6^-methyladenylated GGAC motif [11], while Drosha cuts the pri-miRNA duplex at its hairpin structure [12]. Processed pre-miRNAs are then exported from the nucleus to the cytoplasm by the exportin 5 (Xpo5)/RanGTP complex [13,14] (Figure 1A). In the cytoplasm, pre-miRNAs are further processed into mature miRNA duplexes through removing terminal loops by another RNase III enzyme, Dicer [15] (Figure 1A). One of the mature miRNA strands (the guide strand) is then loaded onto Argonaute (Ago) protein and, together with other associated proteins, forms the RNA-induced silencing complex (RISC) [16]. In RISC, the miRNA recognizes its target mRNAs through base pairing between the short (6–8 nt) seed region of the miRNA and miRNA target sites on mRNAs [17,18] (Figure 1A). The RISC then either destabilizes target mRNAs or suppresses the translation of mRNAs, both of which lead to the post-transcriptional silencing [18,19]. Due to the partial complementarity and the short seed sequence, one miRNA is able to modulate the expression of hundreds of genes [20].

In addition to its role in making mature miRNAs, Dicer is also required for the maturation of short interfering RNAs (siRNAs). In eukaryotes, endogenous siRNAs (endo-siRNAs) can be generated from different sources of transcripts that form duplexes [21] (Figure 1B). In contrast to miRNAs, siRNAs are generated independently of microprocessors [22]. Another difference is the higher degree of complementarity between siRNAs and target mRNAs, which activates the slicing activity of Ago2 and leads to the cleavage of target mRNAs (Figure 1B). As a consequence, the silencing effect of siRNAs is usually considered to be stronger than that of miRNAs, although miRNAs can also perform potent inhibitory activities depending on the relative abundance between miRNAs and their targets [23,24]. Even though endo-siRNAs play important roles in heterochromatin formation and gene regulation in lower organisms [24], their functions in mammals are largely unknown and still remain to be investigated.

## 3. Development of Early Embryos in Mice and Humans

### 3.1. Formation of Gametes

In mice and humans, formation and development of germ cell lineages start with the specification of primordial germ cells (PGCs) in early embryos. In mice, PGCs can be identified as a cluster of ~40 cells at the base of the incipient allantois at ~E7.25 (embryonic day 7.25) [25,26]. PGCs then migrate along different compartments to reach genital ridges (precursors of gonads) at ~E10.5 [27,28,29,30], in where PGCs differentiate toward precursors for oocytes (oogenic pathway) or sperm (spermatogenic pathway). The initial step of the formation of oocytes, or oogenesis, is the formation of primary oocytes, which happens before or shortly after birth. After puberty, primary oocytes go through meiosis I to form secondary oocytes and first polar bodies. In vertebrates, secondary oocytes then progress through a part of meiosis II and arrest at metaphase II. After fertilization, meiosis II is completed, forming the mature 1N ovum (egg cell) and the secondary polar body. In both meiosis I and II, daughter cells are segregated in an asymmetric manner, leaving most of the cytoplasmic material in mature oocytes. In contrast, sperms are formed through two symmetric divisions of primary spermatocytes, followed by the generation of tailless spermatids. Spermatids then transform into sperms, losing most of its cytoplasm [31]. Thus, a fundamental difference between oocytes and sperms is the content of cytoplasmic materials. In mammals, a sperm carries only 10–20 fg RNA, while oocytes can carry 0.5–0.7 ng RNA [32]. Despite of the different RNA content, the relatively small amount of RNAs in sperms could still play important roles in fertilized embryos [33].

### 3.2. From Zygotes to Blastocysts

**Figure 2:**
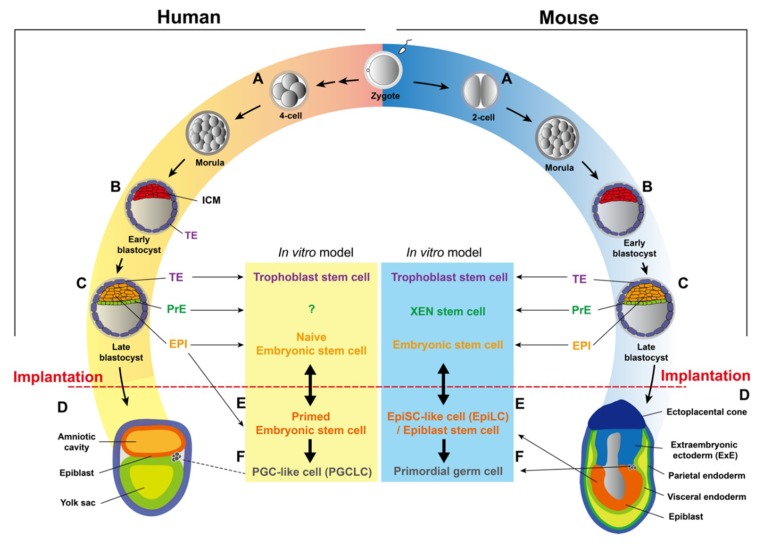
Progression of cell potencies in early embryos and cultured cells. The pre-implantation embryonic development starts with fertilized oocytes, followed by the zygotic gene activation at the 4-cell stage and 2-cell stage blastomeres in humans and mice, respectively (**A**). Zygotes and 2-/4-cell stage blastomeres are totipotent, forming the whole fetus, including embryonic and extraembryonic lineages that will give rise to the yolk sac and placenta in the future. Through the morula stage, the embryonic and extraembryonic lineages are segregated in the early blastocyst, forming the inner cell mass (ICM) and the trophectoderm (TE) (**B**). Pluripotent stem cells are emerged from the ICM and further separated from the primitive endoderm (PrE) to form the epiblast (EPI) in the late blastocyst (**C**). These three main lineages (TE, PrE, and EPI) can give rise to three types of stem cells (trophoblast stem cells [TSC], extraembryonic endoderm [XEN] stem cells, and embryonic stem cells [ESC]) with corresponding cell potencies. In humans, the culture condition for XEN stem cells remains to be discovered. Both EPIs and ESCs are considered to be in the “naïve” or “ground” state and need to be “primed” for further differentiation. In utero, this priming happens after implantation, leading to the reduced pluripotency of the EPI in post-implantation embryos (**D**). In vitro, primed pluripotent stem cells can be directly derived from epiblasts of post-implantation embryos or converted from the naïve ESCs as EpiSC-like cells (EpiLCs) (**E**). Finally, the very first germ cell lineage, primordial germ cells (PGCs), are specified after implantation. Since PGCs are either impractical to be isolated (in humans) or difficult to maintain as the primary culture, they are usually substituted by converting the pluripotent stem cells to PGC-like cells, PGCLCs (**F**). The black dashed line indicates the corresponding cell potency.

In mice, following fertilization, zygotes undergo three cleavages to form 8-cell embryos [34] (Figure 2). One major event, the maternal-to-zygotic transition (MZT), occurs during the first three cleavages. During the MZT, transcription from the zygotic genome is activated, known as the zygotic genome activation (ZGA), to support the subsequent embryonic development [35,36]. In mice, this process is further divided into the minor ZGA, which occurs in the middle S phase of the 1-cell embryo, and the major ZGA, which peaks between the 2-cell and 4-cell stage, characterized by a more extensive genomic reprogramming [37,38,39]. Another crucial event happens during the MZT is the clearance of maternal RNAs, which is triggered by the meiotic maturation of oocytes. By the 2-cell stage, 90% of maternal transcripts are degraded [40]. Failed clearance of maternal transcripts in mice led to the infertility, highlighting the importance of switching transcriptomes during the very early embryonic development [41,42].

Blastomeres of early 8-cell stage mouse embryos are still totipotent, capable of contributing to all embryonic and extra-embryonic cell lineages [43,44,45]. Subsequently, increased intercellular adhesion between blastomeres leads to the compaction, an apical-basal polarization appearing at the late 8-cell stage [45]. After compaction, several rounds of cleavage take place to form early blastocysts, which contain two distinct cell populations: the external layer of the embryo, or the trophectoderm (TE), gives rise to the placenta, while internal cells of the embryo form the inner cell mass (ICM) [34,45,46] (Figure 2B). At E4.5, the ICM subsequently segregates into the epiblast (EPI), which generates future cell lineages of the embryo proper, and the primitive endoderm (PrE), which gives rise to the yolk sac [34,44,45] (Figure 2C).

In humans, the minor ZGA occurs at the 2-cell stage [47,48], while the major wave of ZGA takes place between the 4-cell and 8-cell stage on embryonic day 3 (E3) [49]. Mid-preimplantation gene activation (MGA), peaking at the 8-cell stage, is also a period when maternally inherited RNAs and proteins are degraded [37,38,39]. Following the ZGA, the embryo undergoes compaction to form the morula that marks the first morphological indication of a break in radial symmetry, followed by the formation of the blastocyst between days 5 and 7 post-fertilization (Figure 2B). Despite the delayed timing, mature human blastocysts also consist of three lineages (EPI, PrE, and TE), whose developmental potential is considered to be in parallel to corresponding compartments in mice (Figure 2C).

### 3.3. Development after Implantation

Implantation, or the attachment of embryos to uteri, happens at E4.5 in mice, while human embryos undergo one additional round of cell division before implantation on day 7 [49]. In mice, the implanted embryo further elongates to form a radially symmetric egg cylinder containing the more specified epiblast (EPI) and the visceral endoderm (VE), derived from the PrE, as well as the extraembryonic ectoderm (ExE) and the ectoplacental cone, both of which are derived from the TE [50] (Figure 2D). During further development, the EPI gives rise to three germ layers (ectoderm, mesoderm, and endoderm) of the embryo, the extraembryonic mesoderm of the yolk sac, the amnion, as well as the allantois [51]. The VE will differentiate into the extraembryonic endoderm (visceral endoderm and parietal endoderm), which gives rise to the yolk sac (including visceral yolk sac and parietal yolk sac) [52,53] (Figure 2D). Finally, progenies of the TE, including the ExE and the ectoplacental cone, will generate trophoblast lineages of the placenta [54] (Figure 2D).

## 4. Stem Cells Derived from Early Embryos

Both mouse and human preimplantation embryos are composed of the epiblast (EPI), the primitive endoderm (PrE), and the trophectoderm (TE), which are able to give rise to the three germ layers of the fetus, the yolk sac, and the placenta, respectively [55]. After years of efforts, three in vitro cell culture models have been established to recapitulate potentials of those three distinctive lineages: embryonic stem cells (ESCs), extraembryonic endoderm (XEN) stem cells, and trophoblast stem cells (TSCs) [56,57,58,59,60] (Figure 2). There are two more types of stem cells that can be isolated shortly after implantation: the epiblast stem cell (EpiSC) which recapitulates the potential of the more primed epiblast (Figure 2E and see later), and the primordial germ cell (PGC), the very first precursor of the germ cell lineage [26,61,62] (Figure 2F). it is worthy of note that there are substantial differences in expression profiles of those three lineages of preimplantation embryos between mice and humans [63]. Thus, functions and regulatory mechanisms of those stem cells may not be completely conserved in those two species. Another thing that needs to be mentioned is that derived stem cells could be in a differentiated or de-differentiated state, owing to the signaling cues from the culture medium or feeder layers. In the next section, we will briefly discuss the in vitro models of stem cells in early embryos, particularly the differences between mice and humans, as well as different cell fate potential resulted from culture conditions.

### 4.1. Embryonic Stem Cells

Mouse ESCs (mESCs) were isolated from the ICM or EPI at E3.5 and E4.5 [56,64] (Figure 2). Initially, mESCs were cultured on mitomycin C-treated STO fibroblasts (feeder layers) with growth medium containing calf serum. Later, it was found that supplementing the leukemia inhibitory factor (LIF) inhibits embryonic stem cell differentiation [65]. mESCs recapitulate the pluripotent potential of the EPI and can differentiate into almost all cell types of three germ layers in vitro. Importantly, mESCs can form chimeras while being aggregated with or injected into the morula/blastocyst, demonstrating their pluripotency in vivo [66]. If the microinjection experiment is performed by using the 4N (tetraploid) recipient embryos, mESCs are even able to generate whole-ESC mice, since 4N cells can only form extraembryonic, but not embryonic, tissues, undeniably proving the full pluripotency of mESCs [67].

Human ESCs (hESCs) were first isolated from the ICM of the blastocyst produced by in vitro fertilization [58]. The primitive hESCs are maintained on irradiated mouse embryonic fibroblasts as feeder layers with the growth medium supplemented with fetal bovine serum [58]. Later, researchers demonstrated that a medium supplemented with basic FGF (bFGF) and BMP signaling inhibitor can sustain undifferentiated proliferation of hESCs in the absence of feeder layers or the conditioned medium [68]. Although mESCs and hESCs share similar embryonic origins, there are fundamental differences between mESCs and hESCs: (1) the morphology of hESC colonies is flattened, while mESC colonies are in a dome shape; (2) different pluripotency markers, such as SSEA-3 and SSEA-4, are expressed in hESCs, while SSEA-1 is expressed in mESCs; (3) the self-renewal of hESCs is dependent on FGF/TGFβ signaling pathways, while mESCs use LIF/BMP4 signaling pathways for their self-renewal [58,64,65,68,69,70]. These differences raise a question as to whether mouse and human ESCs are in equivalent states of cell potency. Actually, the morphology and the culture conditions of hESCs more resemble those of mouse epiblast stem cells (EpiSCs) derived from the post-implantation embryo (see later) [71,72], suggesting that hESCs cultured in the conditions mentioned above probably correspond to the EPI of the post-implantation embryo, which is more ready for lineage specification. This cell fate potential is coined the “primed” pluripotency state, in order to distinguish it from the “naïve” pluripotency state of the EPI in the pre-implantation embryo (Box 1).

Box 1 **Culture conditions and pluripotent states of mESCs.**

Efforts to optimize the culture condition for mESCs can be regarded as the process of figuring out signaling pathways for the self-renewal and differentiation of mESCs. For decades, mESCs were mostly cultured with calf serum on the feeder layer, supplemented by leukemia inhibitory factor (LIF). Further studies suggest that the serum can be replaced by bone morphogenetic proteins (BMPs) and the LIF/BMP combination is sufficient to sustain the self-renewal of ESCs [73]. LIF activates the Janus-associated kinase (JAK)/Stat3 pathway, as well as mitogen-activated protein kinase (Erk) cascade [74]. Strikingly, mESCs don’t require the mitogenic Erk signaling pathway for proliferation and, inversely, the Erk pathway actually promotes differentiation, as inhibiting the Erk pathway can sustain mESCs in the absence of LIF for a period of time [75,76]. In addition to the Erk cascade, activation of the Wnt signaling pathway by inhibiting of glycogen synthase kinase 3 (GSK3) also supports mESC self-renewal [77]. Through the combination of the Mek/Erk inhibitor, PD0325901, and the GSK3 inhibitor, CHIR99021, the Smith group figured out a condition (2i) for sustaining mESCs in a defined medium without the need for serum or feeder layers [78]. mESCs cultured in this medium are in a state termed “ground”, or “naïve”, since no external signaling is required for the maintenance of pluripotency, analogous to the EPI in the blastocyst. Although it is not essential, in practice, LIF is combined with 2i (2i/LIF) to promote the colony propagation of ESCs [76]. With the advent of 2i or 2i/LIF culture condition, the conventional serum/LIF/feeder culture is viewed as a condition leading to more heterogeneous mESCs, containing both the ground state population and the cells exited from the ground state [79].

If hESCs are actually in the primed pluripotency state, like mouse EpiSCs, is there a naïve state of hESCs that is the counterpart of mESCs? If there is, what could be the culture condition for it? Since 2010, several breakthrough studies have confirmed the existence of the naïve pluripotency state of hESCs using different approaches [80,81,82,83,84,85,86,87] (Figure 2). Some observations have been made that have indicated the naïve pluripotency of hESCs, such as the dome-like morphology, the activation of specific enhancer for Oct4, X chromosome reactivation (XaXa), higher mitochondrial respiration, expression of specific transposable elements, similar expression profiles compared to morula/early embryos, DNA hypomethylation, and the ability to form interspecies chimeras [80,81]. However, there is still no consensus as to whether all criteria should be applied to assess the naïve pluripotency, especially the chimera assay. It also needs to be noted that the genomic instability could be a problem for current conditions [88,89,90]. In summary, despite the great breakthrough, more studies are needed in order to understand naïve pluripotency in humans.

### 4.2. Extraembryonic Endoderm (XEN) Stem Cells

The extraembryonic endoderm (XEN) (stem) cell is another stem cell line derived from the mouse preimplantation blastocyst or the post-implantation embryo, representing the developmental potential of the PrE [57] (Figure 2). After implantation, the PrE segregates into two subpopulations, the parietal endoderm and the visceral endoderm (Figure 2), which will give rise to part of the parietal yolk sac during the early developmental stage and to the visceral yolk sac during the late developmental stage, respectively [91]. In the chimera assay, XEN cells contribute to both the parietal endoderm and the visceral endoderm, despite a strong tendency toward the former [92]. Different from the skewed potential in vivo, XEN cells can be differentiated into visceral endoderm-like cells in vitro [93,94,95].

Human XEN cells are not established, by any means. Again, this could be due to some fundamental differences in PrEs between mice and humans [96,97]. For example, mouse XEN cells can be converted from mESCs by ectopic expression of master transcription factors of the PrE, such as Gata6 or Sox17 [98]. Meanwhile, overexpression of Gata6 and Sox17 is sufficient to convert hESCs into a XEN-like morphology; such cells, however, cannot be maintained [99], suggesting that a different culture condition could be needed for deriving and culturing human XEN cells.

### 4.3. Trophoblast Stem Cells

Mouse trophoblast stem cells (mTSCs), established 17 years after the derivation of mESC, can be derived from the TE of the E3.5 blastocyst or the extraembryonic ectoderm (ExE) of the post-implantation embryo [60] (Figure 2). In vivo, mTSCs recapitulate the developmental potential of the TE, forming trophoblast lineages in the placenta of the chimeric embryo [60]. Yet, in contrast to well-developed procedures for differentiating mESCs into various cell types in vitro, ways of differentiating mTSCs into specific trophoblast lineages are still incomplete. Two of the most important mouse trophoblast lineages, trophoblast giant cells (TGCs) and syncytiotrophoblasts (SynTs), are able to differentiate from mTSCs. Nonetheless, protocols for generating homogenous TGCs/SynTs, as well as other trophoblast subtypes, such as spongiotrophoblasts, are still lacking. Considering the underestimated importance of placenta defects that can lead to embryonic lethal phenotypes [100], more investigations on mTSCs should be conducted to understand the development of extraembryonic tissues.

Derivation of human trophoblast stem cells (hTSCs) has been even more lagged, possibly due to the different gene expression profiles of TEs between mice and humans [101,102]. hTSCs can be derived from day 7 blastocysts or from first trimester (6–8 weeks) placenta [59] (Figure 2). Different from mTSCs, which rely on Fgf4 and conditioned medium (can be replaced by Tgfβ or activin) for the self-renewal, a much more complicated chemical cocktail is required for the derivation and maintenance of hTSCs [59]. hTSCs can be differentiated in vitro into two major types of human trophoblasts, the extravillous trophoblasts (EVTs) and the syncytiotrophoblasts (STBs) [59]. Even though hTSCs can differentiate into EVTs and STBs, whether it is equivalent to the human TE is still unclear, since cytotrophoblasts (CTBs) of human placenta can also give rise to those two trophoblast lineages. Apparently, more characterization is needed for this new stem cells, such as comparing the expression profile of the hTSC with the human TE, identifying the core regulatory circuitry for its self-renewal, as well as examining its potential to form interspecies chimeras.

### 4.4. Epiblast Stem Cells

Mouse epiblast stem cells (EpiSCs) are derived from the epiblast of the post-implantation embryo [71,72] (Figure 2). EpiSCs cultured in vitro represent the “primed” pluripotent state of epiblast in utero, forming derivatives of three germ layers but rarely incorporating into chimeras following blastocyst injection [71]. As aforementioned, primed hESCs cultured in the canonical condition share a lot of characteristics of mouse EpiSCs, such as morphology, expression of signature genes, signaling pathways for self-renewal, inactivation of one X chromosome (XaXi), and glycolytic metabolism, indicating they are in the same pluripotency state [69].

### 4.5. Primordial Germ Cells

In mice, primordial germ cells (PGCs) arise from post-implantation epiblasts (Figure 2). Primary mouse PGCs isolated from post-implantation embryos can be cultured after reprogrammed by bFGF, Kit ligand, and LIF as the format of embryonic germ (EG) cells, which are not identical to PGCs [103,104,105]. For humans, it is impracticable to isolate PGCs since they are specified at week 2–3 post-fertilization [106]. This difficulty is now circumvented by a breakthrough method that induces PGC-like cells (PGCLCs) from pluripotent stem cells in vitro [107,108,109,110]. In mice, PGCLCs can further differentiate to functional gametes, suggesting they’re functionally comparable to authentic PGCs [107,111]. A similar approach can also induce human PGCLCs, which acquires the germ cell fate [112]. Importantly, PGCLCs can only be differentiated from naïve pluripotent stem cells through an epiblast-like state (epiblast-like cells, or EpiLCs), a route recapitulating the specification of PGCs in utero [107,113] (Figure 2).

## 5. Roles of microRNAs in Early Embryos

### 5.1. Expression of miRNAs in Early Blastomeres

After fertilization, zygotes receive cytoplasmic materials from both sperms and oocytes. As mentioned above, most RNAs, including miRNAs, come from oocytes. The expression of miRNAs in mature oocytes and zygotes is similar, suggesting that zygotic miRNAs are mainly maternally inherited [114,115]. Whether specific miRNAs from sperms play important roles in zygotes for the subsequent development remains an open question. For example, two miRNA clusters, *miR-34b/34c* and *miR-449a/b/c*, are highly enriched in sperms but absent in oocytes [116]. Inhibition of *miR-34c* by injecting antagonistic linked nucleic acids (LNAs) into zygotes led to attenuated first cleavage of zygotes, suggesting that paternal *miR-34c* could participate in embryo development [117,118]. However, genetic ablation of *miR-34b/c* and *miR-449a/b/c* in mice draws a more complicate picture: knocking out both *miR-34b/c* and *miR-449a/b/c* (dKO) led to a severe defect of spermatogenesis. Yet, the dKO round spermatids, while being injected to oocytes, were able to fertilize oocytes and to support the normal embryo development, suggesting that deficiency of *miR-34/449* only affects the development of sperms (the formation of tails) but does not influence the development of fertilized zygotes [116,118]. The discrepancy between those two experiments could be due to the off-target effect of LNAs. Even though *miR-34b/c* and *miR-449a/b/c* are dispensable for the development of mouse embryos, the *miR-34c* level in spermatozoa is corrected with the outcome of intracytoplasmic sperm injection (ICSI), suggesting *miR-34c* could be beneficial for the development of human embryos [119]. Moreover, besides of the canonical inhibitory mechanism through mRNA destabilization, paternally inherited miRNAs have been shown to play important roles in the epigenetic inheritance of zygotes [120,121].

**Figure 3:**
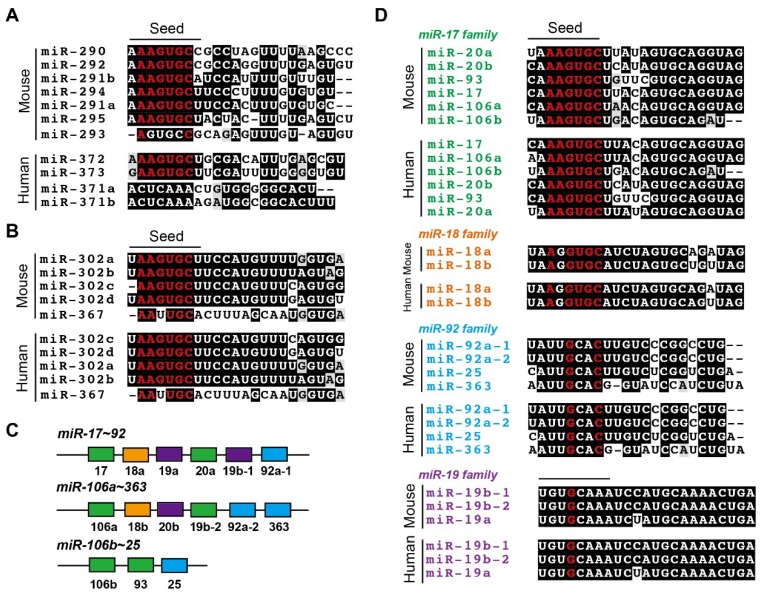
Major miRNA clusters expressed in embryonic stem cells. (**A**) Mouse *miR-290~295* cluster and human *miR-371~373* are homologous miRNAs. Except for *miR-293* in mice and *miR-371* in humans, all miRNAs contain the “AAGUGC” motif in seed sequences (marked in red). (**B**) The sequence alignment of mouse and human *miR-302~367* clusters, which are highly conserved and also contain the “AAGUGC” motif. (**C**,**D**) The structure and sequence alignment of *miR-17~92, miR-106a~363*, and *miR-106b~25* clusters. Please note that the *miR-17* family contains the full “AAGUGC”, while the *miR-18, -19*, and *-92* families only contain a part of the “AAGUGC” motif.

After fertilization, the expressions of many miRNAs (mostly maternally inherited) are down-regulated more than two-fold during the oocyte-to-1-cell transition and the minor ZGA [39]. The most drastic change of total miRNA levels happens during the MZT, when total amount of miRNA is down-regulated by 60% [114]. The degradation of miRNAs is significantly slowed down since the MGA [39], suggesting that the de novo synthesis of miRNAs takes place between the 2-cell and 4-cell stage. Using a novel high throughput microarray assay, Yang and colleagues discovered 67 differentially expressed miRNAs classified into four stage-dependent groups: 7 miRNAs in oocytes, 7 miRNAs in 2-cell blastomeres, 25 miRNAs in 8-cell morulae, and 28 miRNAs in blastocysts [122]. The most abundant maternal miRNAs in zygotes are the *let-7* and *miR-17~92* miRNAs, whose expression are elevated during oogenesis and then inherited by zygotes [114]. The expression of *let-7* and *miR-17~92* is increased again after the 2-cell embryo stage in mice, correlating with the de novo biogenesis of miRNAs [114]. However, the most extensively up-regulated miRNAs in 4-cell blastomeres are the *miR-290* miRNA cluster, *miR-290~295* [123], whose expression is increased 15-fold and 24-fold at the 4-cell and 8-cell stage, respectively, compared to the 2-cell stage [114] (Figure 3). In humans, the majority of miRNAs detected in human oocytes are inherited by zygotes and significantly down-regulated in blastocysts, such as *miR-31*, *miR-16*, *let-7a*, *miR-145*, *miR-210*, and *miR-212* [124,125]. One of the most up-regulated miRNAs in human blastocysts is *miR-371~373*, the human homologue of *miR-290~295* cluster in mice [126] (Figure 3).

### 5.2. Functions of miRNAs in Pre-Implantation Embryos

Although global and specific changes of miRNA expression profiles seem to suggest their functional roles in embryo development, it is surprising that miRNAs may be dispensable for the embryonic development, at least before implantation [127,128,129]. General approaches to study functions of miRNAs is to deplete miRNA biogenesis proteins, such as Dicer, Drosha, or Dgcr8 (Figure 1). However, in pre-implantation embryos, depleting miRNAs in gametes and zygotes exhibited different phenotypes. For example, since *dicer^−/−^* mice are embryonic lethal [128,129,130], zygotic *dicer^−/−^* mice can only be made from intercrossing *dicer^+/-^* mice. In this crossing, *dicer*-deficient oocytes, which are generated from *dicer^+/−^* oogonia, will still contain Dicer in the cytoplasm, and this maternal Dicer will be inherited by *dicer^−/−^* zygotes, even though there will be no de novo synthesis of Dicer anymore. To completely deplete inherited Dicer in zygotes, a maternal knockout approach (for instance, using the Zona pellucida glycoprotein 3 promoter (*Zp3*)-Cre line expressing Cre in developing oocytes) is often employed [114,131,132]. For Dicer, the zygotic knockout resulted in the embryonic arrest at E7.5, with the normal blastocyst formation at E3.5, suggesting that the de novo synthesis of miRNAs is dispensable for blastocyst formation [128,129,130]. On the other hand, maternal depletion of Dicer in oocytes resulted in the meiotic arrest of oocytes with defects in spindle and chromosomal segregation [114,131,133] (Box 2). The maturation defect of oocytes in the *dicer*-deficient mouse model makes it difficult to study maternally inherited miRNAs in zygotes [114,131,133]. Eventually, the functional role of maternally inherited miRNAs was addressed by the maternal *dgcr8* knockout: *dgcr8*-deficient oocytes mature normally and, when fertilized by *dgcr8*-deficient sperms (maternal-zygotic *dgcr8^−/−^* or MZ *dgcr8^−/−^* zygotes), are able to develop into normal blastocysts with the proper segregation of the TE and ICM identified by marker staining [133]. Although the observation above seems to suggest that Dgcr8-dependent miRNAs are dispensable for segregation of the EPI, PrE, and TE lineages, stem cells derived from those lineages exhibit defects in either self-renewal or differentiation, indicating possible discrepancies between in vitro and in vivo models [128,134,135,136,137] (see later). Since the segregation of three lineages in blastocysts is only confirmed by lineage markers, more detailed analyses, such as comparing expression profiles of three lineages between wildtype and MZ *dgcr8^−/−^* blastocysts, will help to decide if there is any defect at this stage. Also, it needs to note that some miRNAs are generated through Dgcr8- or even Dicer-independent pathway [22,138,139] (Figure 1A), and their possible roles in early embryonic development cannot be excluded. Nonetheless, despite of these caveats, current evidence tends to argue that miRNAs, either inherited or de novo synthesized, are not essential for the development up to the blastocyst stage.

Box 2 **Function of Dicer in mouse oocytes.**

Transposable elements (TEs) make ~40% of mammalian genomes. During gametogenesis, extensive epigenetic reprogramming, such as DNA demethylation, happens, leading to the reactivation of TEs. To maintain genomic integrity, mechanisms defending the genome of the germline are evolved by the host. One of them is a mechanism dependent on a class of small RNAs, PIWI (P-element-induced wimpy testis)-interacting RNAs (piRNAs) [140]. Generated through a Dicer-independent mechanism, piRNAs encoding TE sequences associate with PIWI proteins to silence TEs through degrading TE transcripts and/or silencing genomic loci of TEs [141,142]. In male mouse germline, three PIWI proteins, Mili, Miwi, and Miwi2, are expressed, and all of them play essential roles in spermatogenesis [143,144,145]. Knocking out PIWI proteins led to aberrant activation of TEs, causing spermatogenic arrest [146]. Interestingly, the piRNA pathway is dispensable in female mouse germline, possibly due to the lack of the fourth PIWI family member, PIWIL3, which exists in the oocytes of other mammals [147]. An alternative to compensate for the loss of the piRNA pathway and to silence TEs is the endo-siRNA pathway (Figure 2). It has been demonstrated that deficiency of Dicer in mouse oocytes leads to a decreased level of endo-siRNAs and upregulation of TEs [148,149]. Interestingly, an alternative isoform of Dicer, Dicer^O^, is specifically expressed in oocytes, instead of the one expressed in the somatic cell, Dicer^S^ [150]. Dicer^O^ is more efficient at processing endo-siRNAs than miRNAs, and specifically knocking out *dicer^O^* is sufficient to phenocopy the MZ *dicer* knockout, leading to meiotically arrested oocytes with spindle defects [150]. Since *dicer^o−/−^* oocytes only exhibit minor defects in the miRNA expression, and the miRNA activity is suppressed in mouse oocytes [150], the loss of endo-siRNAs could be responsible for the knockout phenotype. However, this doesn’t exclude the possibility that miRNAs may play roles in silencing TEs at later stages of embryonic development.

### 5.3. Functions of miRNAs in Post-Implantation Embryos

In contrast to pre-implantation embryos, roles of miRNAs in post-implantation embryos are more evident: zygotic depletion of *dgcr8* or *dicer* in mice led to growth arrest between E6.5 and E7.5 [129,136]. In implanted zygotic *dicer^−/−^* embryos, the initial specification of the epiblast is normal compared with wildtype ones, despite the expression of *miR-290* miRNAs (*miR-291-3p*, *miR-295*, and *miR-291-5p*) was not compromised in *dicer^−/−^* embryos for unknown reasons [128]. Defects of *dicer^−/−^* embryos only become prominent at the gastrulation stage, in the elongation of the primitive streak and in the specification of definitive endoderm [128]. Those defects are in parallel with differentiation defects in *dicer-* or *dgcr8*-deficient ESCs (see below). It remains to be decided whether defects in embryonic development happen during the transition from the naïve to the primed state of EPI, or during the lineage specification from a normally primed epiblast. Since Dicer controls the biogenesis of both miRNAs and endo-siRNAs, *dgcr8* knockout will be a more appropriate model to perform more detailed analyses. Moreover, since the *miR-290* cluster is still expressed in *dicer^−/−^* embryos, double knockout of *dicer* and *miR-290* may elucidate the role of miRNAs in post-implantation embryos.

In addition to the essential roles of miRNAs in epiblast development, miRNAs are also required for the development of extraembryonic lineages. In *dicer^−/−^* post-implantation embryos, the number of trophoblast stem cells in the ExE, the derivative of the TE lineage, was reduced at E6.5 [128]. Also, the patterning of the VE, the derivative of the PrE lineage, was also severely compromised [128]. Importantly, conditional knockout of *dicer* in the epiblast using the *Sox2Cre* line extended the survival of post-implantation embryos up to E9.5 with normal extraembryonic tissues, suggesting that defects of extraembryonic lineages in post-implantation embryos are not due to defects in epiblast development. Contrarily, defects in extraembryonic lineages could be among the causes for the failure of primitive streak elongation [128]. At this stage, apoptotic cells are greatly increased in *dicer^epi−/−^* embryos, suggesting the main role of miRNAs (or endo-siRNAs) in embryonic tissues could be protecting the cells from dying upon specification [128]. In addition, these results also highlighted the importance of miRNAs in the specification of extraembryonic lineages in utero. With the advent of the era of single-cell transcriptomics, tracing defects of various cell subtypes in *dicer^−/−^* or *dgcr8^−/−^* embryos, including both embryonic and extraembryonic lineages, will help to address roles of miRNAs in the development of post-implantation embryos.

Knocking out *dicer* or *dgcr8* leads to, in most cases, the most severe phenotype since the majority of miRNAs were ablated. In *dicer-* or *dgcr8*-deficient mouse model, it is usually a hard task to figure out which miRNA(s) is responsible for a specific phenotype. Actually, most miRNA-knockout mouse strains are either viable or without obvious phenotypes [7] (Table 1). The *miR-290* cluster is the best guess, since it is highly upregulated during the early embryonic development and is also highly enriched in mESCs [123,151] (Figure 3). Surprisingly, knockout of *miR-290* only resulted in partially embryonic lethality after E8.5 [126] (Table 1). Around 50~60% of *miR-290^−/−^* embryos exhibit two types of developmental defects, the localization of embryos outside of the yolk sac, as well as delayed development (e.g., fewer somites) of embryos [126]. Considering the delayed phenotypes compared with *dicer^−/−^* or *dgcr8^−/−^* embryos, it cannot be excluded that defects of *miR-290^−/−^* embryos could be due to the abnormal development of extraembryonic tissues. Actually, a recent study demonstrated that *miR-290* is highly expressed throughout embryogenesis from E2.5 to E6.5, disappears in the embryo proper at E7.5, but remains highly expressed in the yolk sac and placenta [152]. Knocking out *miR-290* resulted in the loss of trophoblast progenitors, the reduced-size placenta, and the defect in the maternal-fetal transport [152] (Table 1). Taken together, those results suggest that *miR-290* deficiency recapitulates phenotypes of *dicer*/*dgcr8* knockout more in extraembryonic tissues, rather than in the epiblast. Since the development of embryos and extraembryonic tissues are coordinated, tissue-specific knockouts in extraembryonic lineages will be helpful to clarify the complexity.

### 5.4. miRNA Activities during Early Development

Why are miRNAs, no matter whether inherited or de novo synthesized, present but do not seem to be functional during the early embryonic development until the blastocyst stage? One possibility is that the expression of miRNA biogenesis proteins, such as Drosha, Dgcr8, Xpo5, and Dicer, are drastically downregulated during zygotic cleavages and their expression levels remain low until the blastocyst stage [164]. The lack of miRNA biogenesis explains why zygotic knockout of *dicer* or *dgcr8* does not exhibit phenotypes up to the blastocyst stage since the biogenesis of miRNAs is already low even in wildtype embryos. However, low expression levels of miRNA biogenesis proteins may not be the rate-limiting factor for miRNA maturation, since some miRNAs, such as the *miR-29b* and *miR-290* clusters, have been found to be expressed at the 2-cell, 4-cell, and 8-cell stage [114,165]. A complete survey of 238 miRNAs during early development of mouse embryos also revealed several miRNA expression patterns and some miRNAs are up-regulated at the early embryonic stage [118]. In summary, it is still unclear about the amount and dynamic of miRNAs at each embryonic stage. A landscape of miRNA expression profiles at each stage of early embryonic development will be crucial to addressing this question.

The following question is, even if mature miRNAs are made in early embryos, are they functional? In other words, does the miRNA effector machinery work in early embryos? Interestingly, there is clear evidence demonstrating that the miRNA activity is strongly suppressed in mouse oocytes, since wildtype and *dgcr8^−/−^* oocytes exhibited basically identical mRNA expression profiles, and miRNA activity is low in oocytes, as demonstrated by the miRNA reporter assay [132,133]. Suppression of miRNA activity is attributed to the expression of an alternative Ago2 isoform, which lacks all known functional domains [166]. The inhibitory effect might be relieved after the transcription of normal *ago2* at the 4-cell and 8-cell stage. Thus, examining miRNA activities at different embryonic stages using reporters will help to clarify this issue [132].

## 6. Functions of miRNAs in Stem Cells

Two important features of pluripotent stem cells are self-renewal and differentiation. Regular in vitro differentiation methods for testing the potency of pluripotent stem cells includes the withdrawal of LIF and providing cues for the specification toward specific germ layers and their progenies. However, with more understanding of different states of pluripotency, the roles of miRNAs in pluripotent stem cells should be addressed with more cautiousness. For example, differentiation defects of ESCs could be due to (1) the naïve-to-prime transition of ESCs, (2) defects in entering/exiting an intermediate state such as the formative state [79], or (3) the lineage specification process (differentiation) of primed ESCs. Indeed, recent evidence suggests that some differentiation defects of ESCs could be due to the naïve-to-prime transition. Here, we discuss recent findings on functions of miRNAs in ESCs of different pluripotent states, in TSCs, in XEN cells, and in PGCs.

### 6.1. Functions of miRNAs in the Expanded Pluripotency State

Conventional pluripotent stem cells, by definition, are only capable of generating the embryonic portion, forming derivatives of three germ layers. However, blastomeres at the earlier embryonic stage, such as the 2-cell stage in mice, exhibit a totipotent potential, giving rise to both embryonic and extraembryonic tissues [167]. So far, no in vitro cell culture models recapitulate the “authentic” totipotency of early blastomeres in vivo, which, by themselves, are able to give rise to the whole embryo. Yet, several recent studies indicate that pluripotent stem cells can be converted to and stably maintained in a state with expanded potential and, while being injected into early mouse embryos, are able to form both embryonic and extraembryonic tissues in chimeras [168,169,170,171]. Choi and colleagues found that knockout of *miR-34* in embryonic stem cells resulted in a “bi-directional” cell fate potential, giving rise to both embryonic and extraembryonic lineages in chimeras [168]. Subsequent studies suggested that *miR-34* blocks the transition from the pluripotency state toward the bi-directional state [168]. Deficiency of *miR-34a* led to the activation of the MuERV-L endogenous retroviruses, a feature shared by totipotent 2-cell blastomeres [168]. Interestingly, the level of *miR-34* also increases during the formation of blastocysts, suggesting that *miR-34* may also restrict the totipotency and play a role in the segregation of the EPI/PrE/TE lineages in vivo [168]. Since the development and the formation of chimeras could be two different processes, more studies are needed to elucidate the role of *miR-34* in the development of early embryos.

### 6.2. Functions of miRNAs in the Naïve Pluripotency State

Mouse ESCs represent the naïve cell fate potential of the ICM/EPI. However, there are differences between in vivo and in vitro model [134,136]. Both *dicer^−/−^* and *dgcr8^−/−^* ESCs maintain the normal ESC morphology, express pluripotent marker genes, but exhibit defects in proliferation due to the G1 phase accumulation [134,136]. In contrast, no obvious defects were observed in *dicer^−/−^* post-implantation embryos until E5.5, suggesting there are no discernible defects in the proliferation and differentiation of epiblasts until then [128]. Also, while differentiating into embryoid bodies (EBs), *dicer^−/−^* ESCs failed to express either endodermal (*hnf4*) or the mesodermal (*brychyury*, *bmp4*, and *gata1*) markers [134]. In contrast, careful examination of embryos with whole-body or epiblast-specific depletion of *dicer* revealed that there are no defects in the induction of *brychyury* (the initial formation of the primitive streak) or the formation of visceral endoderm [128]. In aggregate, there are evident differences in both cell proliferation and differentiation between pluripotent stem cells in vitro and in vivo. There are several explanations for the differences between *dicer*- and *dgcr8*-deficient ESCs and embryos. First, the maternal Dicer could compensate the loss of zygotic Dicer, enabling the *dicer^−/−^* epiblast to proliferate and differentiate to certain extend. Second, both *dicer^−/−^* and *dgcr8^−/−^* ESCs are difficult to obtain since the escape from the initial proliferation arrest may be required for the derivation [134,136]. Hence, some properties of *dicer^−/−^* or *dgcr8^−/−^* ESCs could be altered during derivation and are no longer identical to the *dicer^−/−^* or *dgcr8^−/−^* EPI. Third, in vitro differentiation conditions are likely not able to recapitulate the elegantly controlled signal sequences during the development. Even though the roles of miRNAs in *proliferation* seem to be different in vitro and in vivo (despite the complexity due to maternal Dicer), the lethality of *dicer^−/−^* and *dgcr8^−/−^* embryos at the gastrulation stage indicates that miRNAs do participate in the post-implantation *development*, which can be studied using *dicer^−/−^* or *dgcr8^−/−^* ESCs as differentiation models.

The miRNA profiling has been performed to study the function of miRNAs in mESCs [171]. Two miRNA clusters are dominantly expressed in naïve mESCs: *miR-290~295* and *miR-17~92* [172] (Figure 3A,C,D). Two paralogs of *miR-17~92*, *miR-106a~363* and *miR-106b~25*, are also expressed in mESCs at the lower level [171] (Figure 3C,D). *miR-290~295*, a polycistronic miRNA containing six miRNAs, harbors the seed sequence AAGUGC which is also shared by another miRNA cluster, *miR-302~367*, which is also expressed in mESCs [173] (Figure 3B). Consistent with the same potential state, the human analog of *miR-290~295*, *miR-371~373*, is also highly expressed in naïve hESCs [174] (Figure 3A). Surprisingly, despite their high abundance, single knockout of *miR-290~295* or double knockout of *miR-290~295* and *miR-302~367* in mESCs does not influence the expression of pluripotency genes, suggesting that knocking out those miRNAs does not phenocopy *dicer* or *dgcr8* knockout that compromises the self-renewal of mESCs [126,175] (Table 2). It remains to be studied whether human *miR-371~373* and/or *miR-302~367* are also dispensable for the pluripotency or proliferation of naïve hESCs. Interestingly, in mice, triple-knockout of three other miRNAs (*miR-17~92*, *miR-106a~363*, and *miR-106b~25*) with the abundance secondary to *miR-290~295* exhibits no effect on the embryonic development until E15 [153] (Table 1). Despite the laborious work, knockout of multiple miRNAs, including the *miR-290* family and the *miR-17~92* family, will help to answer whether there is functional redundancy of these miRNAs.

Another miRNA cluster, although it is not highly expressed, is actually important in regulating the stemness of ESCs. *let-7* family miRNAs are highly redundant, containing 10 subfamilies distributed at 13 loci in mice and humans. Expression of mature *let-7* is low in mESCs due to the antagonistic effect of Lin28. Lin28 proteins, including Lin28a and Lin28b, selectively bind to the loop of pre- and pri-*let-7* and block their processing by Dicer and Drosha, respectively [186,187,188,189]. On the other hand, *let-7* also targets *lin28a* and *lin28b*, forming negative feedback loops [190]. In general, *let-7* is highly expressed in somatic tissues but absent in many types of stem cells, suggesting its role in promoting differentiation [180,191,192]. Overexpression of *let-7* suppressed the self-renewal of *dgcr8^−/−^* ESCs, but not wildtype ESCs alone (with normal *miR-290* expression) or *dgcr8^−/−^* ESCs that co-introduced with *miR-290* miRNAs, suggesting that *let-7* and *miR-290* miRNAs play opposing roles in maintaining the self-renewal of mESCs [180] (Table 2). Subsequent analyses indicate that *let-7* targets hundreds of genes within the pluripotency network, including *sall4*, *nmyc*, *lin28*, and many other targets that are indirectly positively regulated by *miR-290* miRNAs [180]. Thus, decreased *miR-290* and increased *let-7* could be essential for the exit of the naïve pluripotency state.

### 6.3. Functions of miRNAs in the Primed Pluripotency State

There are two aspects to consider with regard to roles of miRNAs in pluripotent stem cells in the primed state: the specification of the primed state, and the differentiation from the primed state. For the former, it has been demonstrated that mouse EpiSCs can be converted from naïve ESCs and this in vitro model recapitulates the naïve-to-primed transition in utero [193] (Figure 1). A modified condition is also able to convert naïve ESCs to epiblast-like cells (EpiLCs), which are in an intermediate state between the naïve (ESC) and the primed (EpiSC) cell potential [107,194,195] (Figure 1). *dgcr8^−/−^* ESCs were significantly less efficient to be converted to EpiLCs, suggesting miRNAs are important for priming naïve ESCs [175]. Surprisingly, double knockout (dKO) of two miRNAs expressed abundantly in ESCs and EpiSCs, *miR-290~295* and *miR-302~367*, respectively, does not influence the efficiency of EpiLC colony formation, but only influences silencing of naïve pluripotency markers and induction of some early post-implantation markers [175] (Table 2). Those different phenotypes between *dgcr8^−/−^* and dKO ESCs during the naïve-to-primed transition suggest other miRNAs also participate in this transition. Recently, a study by Du and colleagues demonstrated a protein involved in the biogenesis of polycistronic miRNAs (such as *miR-17~92*), Isy1, is necessary for the naïve-to-primed transition [196]. A set of miRNAs, including *miR-17~92* and *miR-290*, were up-regulated during the naïve-to-primed transition under the positive regulation of Isy1 [196]. Importantly, overexpression of *miR-20* (one member of the *miR-17~92* family) is sufficient to rescue the inability of *dgcr8^−/−^* ESCs to be converted to the primed state [196]. Taken together, these results suggest that the naïve-to-primed transition could be controlled by the cooperation of multiple families of miRNAs. it is noteworthy to mention that EpiLCs may not represents all aspects of EpiSCs. For example, a strong cell death is observed in EpiLCs 48 h after Fgf2/activin induction while EpiSCs can be maintained well with same factors [107]. Thus, EpiSCs converted from ESCs, as well as *dicer^−/−^* or *dgcr8^−/−^* EpiSCs either derived from the post-implantation embryo or generated through genome editing, will elucidate the function of miRNAs in the establish, maintenance, and differentiation of/from the primed state.

As mentioned previously, *let-7* plays an important role in the differentiation of mESCs from the naïve state [180]. Again, this effect could take place during the naïve-to-primed transition or during the differentiation from the primed state. A recent study addressed this question by demonstrating that knockdown of *lin28* in mESCs delayed the naïve-to-primed transition while overexpression of *lin28* exhibited the opposite effect [197]. Following studies suggest that Lin28 regulates the naïve-to-primed transition by targeting the *let-7*-*dnmt3a/b-dppa3* axis [197]. Interestingly, only Lin28a, but not Lin28b, is up-regulated during the naïve-to-primed transition, suggesting those two isoforms play different roles in the exit of the naïve state [197].

Compared to mice, the expression and the function of miRNAs in human embryos are still unclear. Recent studies using cell culture models showed both similarity and disparity in mice and humans. In mESCs, although both *miR-290~295* and *miR-302~367* clusters are enriched, the former is dominantly expressed in the naïve state [175,198]. In contrast, *miR-302~367* are dominantly expressed in EpiLCs and EpiSCs, making itself a characteristic maker for the primed pluripotent state [199]. Consistently, *miR-371~373* (the analog of mouse *miR-290~295*) and *miR-302~367* are dominantly expressed in the naïve and primed hESCs, respectively [174,197] (Figure 3). Ectopic expression of *miR-290~295* miRNAs in mice or *miR-302~367* miRNAs in humans enhances the acquisition of induced pluripotency during somatic reprogramming toward induced pluripotent stem cells [198,200]. Thus, in addition to the expression profile and the shared seed sequence (hence, similar or identical targets), functions of those miRNAs could also be conserved.

Despite comparable expression profiles of miRNAs in the naïve and the primed state, a loss-of-function study suggests that miRNAs could function differently in mouse and human pluripotent stem cells. Teijeiro and colleagues found that directly knockout *DICER1* in human hESCs is not possible [173]. Instead, *DICER1* is absolutely essential for the self-renewal of primed hESCs and can only be depleted transiently [173]. This result in human ESCs is different from mouse ones since *dicer^−/−^* ESCs can still propagate despite of G1 accumulation [135]. The authors then found that *DICER1*-deficient hESCs exhibit no obvious mitotic defects but are prone to apoptosis [173]. Also, knockdown of *DICER* or *DROSHA* in hESCs only led to the slow growth, with the normal expression of pluripotent genes [201]. In aggregate, those results suggest that a basal level of miRNA or endo-siRNA expression is necessary for the survival of hESCs, but not mESCs. Overexpression of either *miR-302~367*, *miR-371~173*, or *miR-17~92* clusters resumed the proliferation of *DICER1*-deficient hESCs, possibly through blocking cell death by targeting the death-receptor *FAS* [173]. Although this transient-depletion system is ideal to address some unanswered questions, such as the role of miRNAs in the naïve state, in the naïve-to-primed transition, and in the differentiation from the primed state, a recent study showed that the biogenesis of some miRNAs is independent on *DICER*, at least in certain human cells [202]. Thus, different models, including *DROSHA* knockout and specific knockouts of miRNAs are still necessary to address functions of miRNAs in hESCs during the naïve-to-primed-to-differentiation process.

### 6.4. Functions of miRNAs in the Cell Potential of the Trophectoderm Lineage

As mentioned above, *dicer^−/−^* mice exhibit strong defects in trophoblast lineages during the development of post-implantation embryos [128]. Consistently, depletion of *dicer* in mouse TSCs led to growth arrest and differentiation toward trophoblast giant cells, suggesting the essential role of miRNAs in the self-renewal of mTSCs [128]. Following studies demonstrate that the *miR-290~295* cluster, in addition to its role in mESCs, is also important for maintaining the proliferation of mTSCs by targeting cell cycle inhibitors such as *p21*, *p57*, *rbl2*, and *lats2* [128] (Table 2). Up-regulation of *p57* in *dicer^−/−^* mTSCs could also promote the endoreduplication and the formation of trophoblast giant cells [128]. It is still unclear whether other miRNAs also play roles in mTSCs.

Since human TSCs were only successfully derived recently, the roles of miRNAs in the self-renewal or differentiation of TSCs remain to be investigated. Notably, one primate-specific miRNA cluster, the C19MC (chromosome 19 microRNA cluster), is expressed in the TE lineage [203]. The C19MC is the largest miRNA cluster in humans, spanning ~100 kb in chromosome 19q13.41 with ~46 miRNAs paternally expressed, especially in the placenta [204]. In the placenta, C19MC miRNAs control the migration and invasion of human trophoblasts in vitro and may elicit an antiviral response in distal tissues during pregnancy while transported by exosomes [205,206]. Some C19MC cluster miRNAs, such as *miR-127* and *miR-372*, are also highly expressed in human TSCs, making it intriguing to know the roles they play in those earliest stem cells of the TE lineage [59]. Besides of the C19MC, an eutherian-specific miRNA cluster, C14MC (within the well-known *dlk1-dio3* locus), is also highly expressed in the human placenta in a maternally imprinted manner [207,208]. Interestingly, C14MC miRNAs do not seem to be expressed in TSCs based on the miRNA-seq result [59]. Knocking out miRNA biogenesis proteins or the C19MC locus in hTSCs will address functions of miRNAs in this newly derived stem cell line.

### 6.5. Functions of miRNAs in the Cell Potency of the Primitive Endoderm Lineage

In vitro, mouse XEN cells recapitulate the differentiation potential of the PrE. Depletion of *dicer* in mouse XEN cells blocked the proliferation and led to up-regulation of downstream lineage markers, including *hex*, *apoe,* and *amot* for the visceral endoderm, as well as *gata4*, *ttr*, *alk2*, and *bpmp2* for the parietal endoderm [128]. Ablation of *dicer* led to the decreased Erk1/2 signaling, suggesting that miRNAs regulates the self-renewal and differentiation through modulating the MAPK pathway in XEN cells [128]. Yet, it is still unclear which miRNA(s) is responsible for this phenotype. In the embryo, the PrE is specified from the ICM and this differentiation process in utero could be recapitulated in vitro by the treatment of retinoic acid/activin or ectopic expression of *gata6* or *sox17* [98,209,210,211,212]. In the future, these two systems can be employed to model the ICM-to-PE transition.

### 6.6. Functions of miRNAs in the Cell Potency of the Primordial Germ Cell Lineage

In mice, *dicer* knockouts are embryonic lethal around the timing of PGC specification [129]. Conditional depletion of *dicer* in epiblasts extended the survival of embryos until E9.5, making it an ideal model for studying the function of miRNAs in the PGC specification [128]. Considering that it is not feasible to study human PGCs in vivo, in vitro models are better choices to study this process. West and colleagues established an in vitro differentiation system to harvest Stella-positive cells in embryoid bodies (EBs) to study the specification of mouse PGCs [184]. In this system, knocking down *lin28* abolished the formation of PGC-like cells while ectopic expression of *lin28* exhibited an opposite effect, indicating *let-7* could involve in PGC specification [184]. Indeed, further studies demonstrated that *let-7* blocks the transition from ESCs to PGCs by targeting *blimp1*, a master regulator for the PGC specification, and this suppressive effect can be relieved by up-regulation of *lin28* [184]. Importantly, these results in vitro can be recapitulated in mouse embryos [184], indicating that this in vitro model is a useful tool for investigating PGC specification. In addition to this EB-differentiation protocol, a procedure for differentiating PGCLCs from naïve ESCs has been established in both mice and humans [112,113] (Figure 2). Importantly, mouse PGCLCs are able to form functional gametes that can produce offspring [107,111]. A recent study employed this cell culture model and demonstrated the role of *miR-372* and *let-7* in human PGC speciation, showing that *miR-372* promotes while *let-7* inhibits the PGC specification [185]. Obviously, more studies employing this system will increase our understanding for roles of miRNAs in critical events of the early PGC specification.

## 7. Discussion

There are two issues that should be considered when exploring functions of miRNAs in the early embryonic development. First, it is necessary to distinguish miRNAs and the miRNA biogenesis machinery that is maternally inherited from that which is de novo synthesized after fertilization. Since *dicer* knockout leads to the meiotic arrest of oocytes, a phenotype that is more attributed to loss of endo-siRNAs [150], the *dgcr8* knockout mouse model serves as an alternative model to study miRNA functions since *dgcr8^−/−^* oocytes are functionally normal [133]. Yet, the other complexity lies in the biogenesis process of miRNAs. Even though the generation of most miRNAs are dependent on the microprocessor (Drosha/Dgcr8) and/or Dicer, some non-canonical miRNAs indeed exist. For example, mirtrons are generated in the *dgcr8*-dependent, *dicer*-dependent manner [22] (Figure 1). Moreover, a recent study in humans suggests that 5p miRNAs can be generated without Dicer [204]. Actually, there are different impacts on the miRNA biogenesis upon the knockout of *DROSHA*, *DICER*, or *XPO5* in human cells [202]. Thus, careful analysis of expression profiles of miRNAs in different knockout models will be necessary to address miRNA functions in the early embryonic development.

The second difficulty in investigating roles of miRNAs in early embryogenesis is the functional redundancy of miRNAs. In mammals, a miRNA family containing similar or identical seed regions could consist of several individual miRNAs located at different chromosome loci and can compensate for the loss of other family members. This functional redundancy of miRNAs is best exemplified by *miR-34/449* family which consists of six homologous miRNAs located at three genomic loci [158]. Mice with two-and-half loci (two alleles for each locus) depleted only exhibit a weak phenotype, whereas complete knockout of three loci led to postnatal mortality due to defects in ciliogenesis [158]. With the advent of CRISPR/Cas9-based genome editing technologies, knocking out all homologous miRNAs could reveal unexpected roles of miRNAs in the embryonic development or the stem cell potential.

In vitro cultured stem cells are valuable models for studying their cell potential in vivo. Cell culture models are particularly important for studies in humans, especially for the stem cells appeared at the later stage, like PGCs. In vitro cultured human embryos can be extended to 12–13 days [213,214], shortly after the first detection of PGCs in human embryos at day 11 [215]. Due to the ethical 14-day limit for culturing human embryos, only the very early speciation of PGCs can be studied with this system. The in vitro differentiation system of ESCs toward germ cells through PGCLC intermediates therefore provides a unique advantage to overcome the availability of human embryos and the scarce of PGCs. Same advantages can be applied to other in vitro cultured stem cells from human early embryos. However, three potential caveats should be kept in mind while using them to investigate roles of miRNAs. First, the genomic instability of ESCs cultured in the naïve state due to MEK inhibition [89], which might be resolved by a modified culturing condition, although more extensive characterization is still needed [88]. Second, the culture conditions for human TSCs have only recently been discovered, and the potency and property of hTSCs remain to be elucidated [59]. Third, the culture conditions for human XEN stem cells is not identified yet. Considering the fundamental difference in expression profiles between mouse and human blastocysts [63], it is reasonable to expect some disparities between mouse and human XEN cells in both regulatory circuitries and cell potencies.

With more understanding of miRNAs in stem cells, it is possible to alter their cell fate potential by manipulation at the level of specific miRNAs. For example, *miR-290~295* and *miR-302~367*, both of which are enriched in ESCs, are able to promote the somatic reprogramming, i.e., the generation of iPSCs [8,200]. Also, in mice, deficiency of *miR-34* led to the expanded cell fate potential toward both embryonic and extraembryonic lineages [168]. Hence, it is intriguing to see if inhibition or overexpression of certain miRNAs can drive stem cells cross barriers of the EPI, PE, and TE lineages in mice and in humans, an area not fully explored yet. Moreover, since sperm *miR-34c* correlates with the successful rate of ICSM, manipulating the activity of *miR-34c* in human zygotes could also be beneficial for in vitro fertilization [119]. Thus, with more extensive investigations, miRNAs could contribute to clinical applications in the future.

In summary, although there has been substantial evidence for the involvement of miRNAs in the development and differentiation, we just start to explore functions of miRNAs in stem cells of early embryos, especially in humans. With more advances in single-cell omics, culture conditions, and genome engineering technologies, tracing and studying roles of miRNAs in the formation and development of those “ancestor” cells will provide us new insights into secrets of the early stage of life.

## Figures and Tables

**Table 1:** Studies of specific miRNAs in knockout mouse models.

Knockout Mouse Model	Perinatal Phenotype	Phenotype at Embryonic Stages	Target	Ref.
*miR-17~92^−/−^*	Early postnatal lethality and very specific defects in the development of heart, lungs, and B cells	Smaller size of embryos at E13.5 and E18.5; ventricular septal defects in heart at E18.5; severely hypoplastic lungs from E18.5–P0; greatly reduced percentage and absolute number of pre-B cells at E18.5	*bim*	[153]
*miR-17~92^Δ17^, miR-17~92^Δ18^, miR-17~92^Δ19^, miR-17~92^Δ92^, miR-17~92^Δ17,18^ and miR-17~92^Δ17,18,92^*	Depletion of individual or multiple miRNAs in this cluster led to variable phenotypes. Perinatal lethality and lung hypoplasia only in *miR-17~92^Δ17,18,92/Δ17,18,92^* mice; Co-deletion of *miR-17 and miR-18* has significant weight-reduction; *miR-17~92^Δ17/Δ17^* mice show reduction of pre-B-cells.	*miR-17~92^Δ^^1^^7,18/Δ17,18^* mice display fusion of the proximal carpal bones.	*tbx3, tbx20, smad6, heg1, klf2* and *trip11*	[154]
*miR-106b~25^−/−^; miR-17~92^−/−^* double knockout	NA	Embryos die before E15 with much more severe phenotypes compared to embryos lacking *miR-17~92* alone; edema and vascular congestion at E13.5 and E14.5	*bim*	[153]
*miR-17~9^−/−^; miR-106~2^−/−^; miR-106a~363^−/−^* triple-knockout	NA	Embryos die before E15 with much more severe phenotype compared to embryos lacking *miR-17~92* alone	*bim*	[153]
*miR-290~295^−/−^*	Germ cell deficiency in surviving adults	Partially penetrant embryonic lethality; *miR-290~295^−/−^* blastocysts at E3.5 exhibit no obvious phenotype; *miR-290~295^−/−^* embryo were lost between E11.5 and E18.5; at E10.5, about 16% of *miR-290~295^−/−^* embryos were partially or completely localized outside the yolk sac	*NA*	[126]
*miR-290~295^−/−^*	NA	Defects in placental growth; prematurely exit the cell cycle of trophoblast progenitor cells; reduced endoreduplicaton of TGCs; disorganized placenta with the reduced area of vasculature; Reduced diffusional exchange capacity	Multiple (combinationational effect)	[152]
*miR-126^−/−^*	Vascular abnormalities	About 40% of *miR-126^−/−^* mice died embryonically or perinatally with angiogenesis defects	*spred-1*	[155]
*miR-205^−/−^*	Neonatal lethality with compromised epidermal and hair follicle growth	NA	*phlda3* and *inppl1*	[156]
*miR-1~2^−/−^*	Lethality at weaning with dysregulation of cardiogenesis	NA	NA	[157]
*miR-34a^−/−^; miR-34b/34c^−/−^; miR-449^−/−^* triple knockout	60% animals lethal and surviving adults are infertile; respiratory dysfunction	NA	*cp110*	[158]
*miR-302 ^−/−^* and *miR-302* *^−/−^; miR-290 ^−/−^* double knockout	NA	Embryos are normal at E7.5; grossly abnormal in neural development at E9.5; severely abnormal brain development at E13.5; double knockouts arrest prior to neurulation	*fgf15*	[159]
*miR-200b ^−/−^*; *miR-429^−/−^* double knockout	Female infertility	NA	*zeb1*	[160]
*miR-12^−/−-^*	Increased motor activity and fatal epilepsy	NA	*slc6a1, slc1a1, scn2b, scn4b, cacna2d3, cagn2, car7*	[161]
*miR-29ab1^−/−^*	Shorter life span; reduced lymphoid organ cellularity	NA	*t-bet* and *ifn-gamma*	[162]
*miR-133a-1^−/−^; miR-133a-2^−/−^* double knockout	50% perinatal lethality at P0 and P1 with ventricular septal defects; cardiomyopathy and heart failure	Abnormal heart development from E12.5 to E17.5	*srf* and *cyclin d2*	[163]

Abbreviations: NA, not available or not addressed.

**Table 2:** Studies of specific miRNAs in in vitro cell culture models.

Species	Cell	Cell Potential	miRNA	Target	Phenotype	Ref.
Mouse	ESC	Naïve pluripotency	*miR-34a*	*gata2*	*miR-34a* restricts the acquisition of expanded cell fate potential in pluripotent stem cells.	[168]
Mouse	ESC	Naïve pluripotency	*miR-290~295*	NA	*miR-290~295* knockout ESCs are pluripotent and no obvious phenotypes were observed.	[173]
Mouse	ESC	Naïve pluripotency	*miR-290~295*	*casp2, ei24*	Overexpression of *miR-294* rescues the apoptosis phenotype of *dicer^−/−^* ESCs upon genotoxic stress. Deletion of *miR-295* cluster enhances the susceptibility of ESCs toward apoptosis upon DNA damage.	[176]
Mouse	ESC	Naïve pluripotency	*miR-290, miR-183~182*	*miR-290* targets *rbl2*, *cdkn1a*, *lats2*, *mbd2*, etc. *miR-183~182* may have different targets but not elucidated	*miR-294*, *miR-182* and *miR-183* antagonize the differentiation of *dgcr8^−/−^* ESCs induced by *let-7*. *miR-290^−/−^*; *miR-183^−/−^* double knockout ESCs are susceptible for *let-7* induced differentiation.	[177]
Mouse	ESC	Naïve pluripotency	*miR-294/302*	*tgfbr1, tgfbr2, gsk3b*	*miR-294/302* combinatorically suppress epithelial–mesenchymal transition (EMT) and apoptosis in differentiating *dgcr8^−/−^* ESCs induced by *let-7*.	[178]
Mouse	ESC	Naïve pluripotency	*miR-26a, miR-99b, miR-193, miR-199a-5p,* and *miR-218*	NA	Similar to *let-7*, overexpression of these five miRNAs induces differentiation in *dgcr8^−/−^* ESCs	[179]
Mouse	ESC	Naïve pluripotency	*miR-291a/b-3p, miR-294, miR-295*, and *miR-302* (embryonic stem cell specific, or ESCC miRNAs)	*cdkn1a, rbl2, lats2*	Overexpression of ESCC miRNAs promotes cell cycle progression and oppose *let-7*-induced differentiation in *dgcr8^−/−^* ESCs.	[137,180]
			*let-7*	*lin28, cmyc, nmyc, sall4*	Overexpression of *let-7* induces differentiation in *dgcr8^−/−^* ESCs.	
Mouse	ESC	Naïve pluripotency	*miR-320, miR-702* (microprocessor-independent miRNAs)	*p57, p21*	Overexpression of *miR-320* or *miR-702* rescues proliferation defects of *dicer^−/−^* and *dgcr8^−/−^* ESCs.	[181]
Mouse	EpiLC	Primed pluripotency	*miR-302* single knockout, *miR-290* and *miR-302* double knockout	*akt1*	Double knockout of *miR-290/302* leads to the improper transition from ESCs to EpiLCs; overexpression of miRNAs in two clusters rescues the phenotype of *dgcr8^−/−^* ESCs.	[175]
Mouse	EpiSC	Primed pluripotency	*miR-127*	*lefty2*	Inhibiting *miR-127* expression in EpiSCs results in decreasing mesendodermal differentiation.	[182]
Mouse	EpiSC	Primed pluripotency	*miR-20, miR-92, miR-302*	*bim*	Overexpression of either of three miRNAs rescues the apoptosis phenotype of *dicer^−/−^* EpiSCs.	[183]
Mouse	TSC	Extraembryonic	*miR-290~295*	*p21, p57, rbl2,* and *lats2*	Depletion of *dicer* in TSCs results in the differentiation and proliferation block of mTSCs.	[128]
Mouse	XEN	Extraembryonic	*miR-20a, miR-30b*	*rasa2*	Deletion of *dicer* in XEN stem cells leads to differentiation and proliferation block.	[128]
			*miR-669a*	*dusp1*		
Mouse	PGC-like	Germline	*let-7*	*blimp1*	*let-7* blocks the transition of ESCs to PGCs.	[184]
Human	ESC	Primed	*miR-302~367, miR-371~373*	*fas, nik, trailr4,* and *bim*	Overexpression of either of two miRNA clusters rescues the apoptosis phenotype in *DICER^−/−^* ESCs.	[173]
Human	PGCLC	Germline	*miR-372*	*smarcc1, mecp2, cdkn1, rbl2, rhoc,* and *tgfbr2*	*miR-372* promotes PGC specification.	[185]
			*let-7*	*cmyc* and *nmyc*	*let-7* inhibits PGC specification.	[185]
