# Correction: Zhang, Z.; et al. Roles of MicroRNAs in Establishing and Modulating Stem Cell Potential. *Int. J. Mol. Sci.* 2019, *20*, 3643

**DOI:** 10.3390/ijms21113894

**Published:** 2020-05-29

**Authors:** Zhenwu Zhang, Lili Zhuang, Chao-Po Lin

**Affiliations:** School of Life Science and Technology, ShanghaiTech University, 230 Haike Rd, Shanghai 201210, China; zhangzhw2@shanghaitech.edu.cn (Z.Z.); zhuangll@shangaitech.edu.cn (L.Z.)

The authors wish to make the following correction to this paper [1]. The authors regret the incorrect appearance of Figure 1. The figure caption and manuscript discussion of Figure 1 in the original publication are correct. Unfortunately, some items in Figure 1 appear incorrectly colored or misaligned. Therefore, the changes were made for the following reasons:

1. The “Trans” graphic of the endo-siRNA biogenesis pathway: we corrected the misalignment of the line and arrow for indicating the transcription start point. The lower strand of the lower DNA duplex was also lengthened. Colors of the arrows (transcribed strands) were switched to be consistent with the RNA duplexes in the next processing step;
2. The “Inverted” graphic of the endo-siRNA biogenesis pathway: colors of the arrows were switched to be consistent with the RNA hairpin formed in the next processing step. Further, a short line was added for indicating the transcription start point;
3. The “Cytoplasm” part of the endo-siRNA biogenesis pathway: colors of the RNA duplex in the RISC were changed to green and deep pink to be consistent with the duplexes in the previous step.

The mistake was generated during the final editing following the peer-review. The mistake did not affect the review process. Our correction does not change the conclusions of this manuscript.

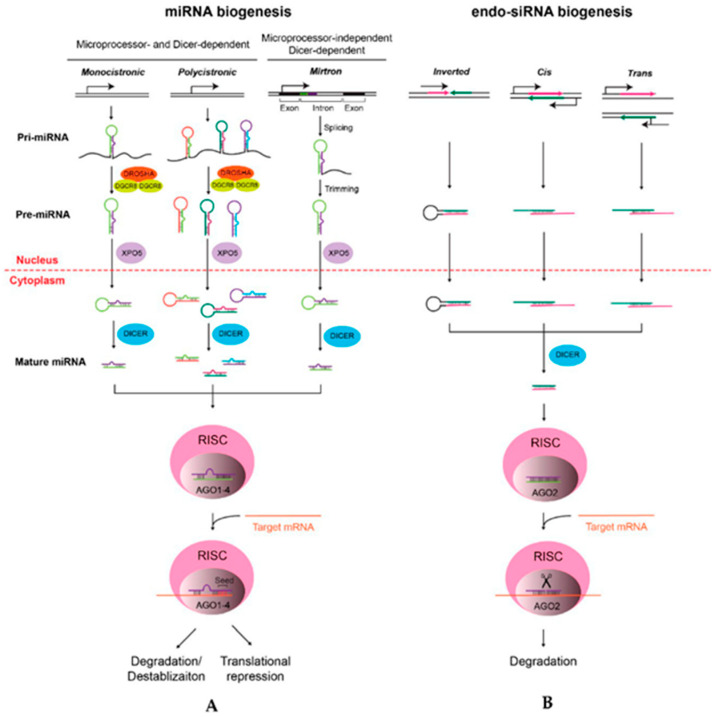



with

**Figure 1:**
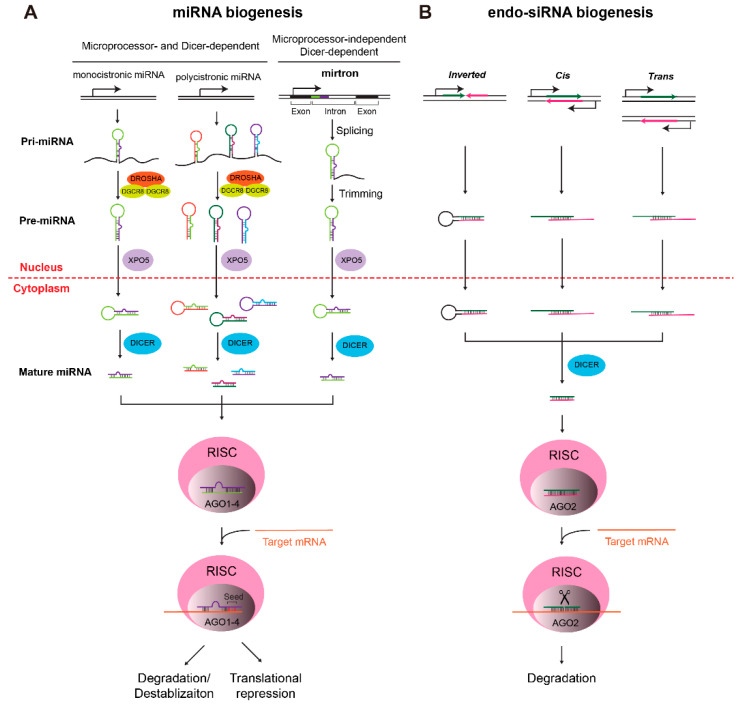
Dicer-dependent biogenesis of miRNAs (**A**) and endo-siRNAs (**B**). (**A**) Biogenesis of miRNAs can be microprocessor- and DICER-dependent (monocistronic and polycistronic miRNAs), or microprocessor-independent and DICER-dependent (mirtrons), with very few exceptions. For the former, monocistronic and polycistronic miRNAs are transcribed by RNA polymerase II as primary miRNAs (pri-miRNAs), which are processed by the microprocessor complex (DROSHA and DGCR8) and exported to the cytoplasm as precursor miRNAs (pre-miRNAs) for the further processing by DICER. Mirtrons, in contrast, are located in the intron and are generated by splicing and trimming that do not need the microprocessor complex to form pre-miRNAs. In both cases, pre-miRNAs need DICER for forming short mature miRNA duplexes. One strand of the duplex is then loaded onto the RNA-interference complex (RISC), where the miRNA recognizes its target mRNA through imperfect base pairing, especially the complementation between the short “seed” sequence of the miRNA and its mRNA targets, performing post-transcriptional silencing on target mRNAs through degradation or translational repression. (**B**) The biogenesis of endo-siRNAs starts with the formation of duplexes from one or two transcripts with complementary sequences. Duplexes are exported to the cytoplasm and processed by DICER as well. Different from miRNAs, endo-siRNAs duplex with their target mRNAs with a higher degree of complementation, inducing the splicer activity of Ago2 for the cleavage of mRNAs, leading to its degradation. Please note that miRNA RISC can be formed with Ago1–4, while the RISC for endo-siRNAs contains Ago2.

The authors would like to apologize for any inconvenience caused to the readers by these changes.
